# Digital governance in tax-funded European healthcare systems: from the Back office to patient empowerment

**DOI:** 10.1186/s13584-020-0361-1

**Published:** 2020-01-17

**Authors:** Paola Mattei

**Affiliations:** Department of Political and Social Sciences, University of Milan, Via Conservatorio, 7, 20122 Milan, Italy

**Keywords:** Digital health, Tax-funded health care systems, Health care organizations, Artificial intelligence, Patients’ engagement, Accountability

## Abstract

Digital healthcare promises to achieve cost-efficiency gains, improve clinical effectiveness, support better public sector governance by enhancing transparency and accountability, and increase confidence in medical diagnoses, especially in the field of oncology. This article aims to discuss the benefits offered by digital technologies in tax-based European healthcare systems against the backdrop of structural bureaucratic rigidities and a slow pace of implementation.

Artificial intelligence (AI) will transform the existing delivery of healthcare services, inducing a redesign of public accountability systems and the traditional relationships between professionals and patients. Despite legitimate ethical and accountability concerns, which call for clearer guidance and regulation, digital governance of healthcare is a powerful means of empowering patients and improving their medical treatment in terms of quality and effectiveness. On the path to better health, the use of digital technologies has moved beyond the back office of administrative processes and procedures, and is now being applied to clinical activities and direct patient engagement.

## Slow pace of digital health governance in Europe

In October of 2018, the Department of Health and Social Care in the United Kingdom published a policy paper on the future of digital data and technology, which highlighted the widening gap between the implementation of digital healthcare and the original aspirations for the service. It clearly stated, “the gap between where we are and where we want to be is only getting bigger” [1]. In 2019, the Topol Review (prepared by the NHS) reached similar conclusions and highlighted the divisive nature of Artificial Intelligence (AI) within healthcare [2]. The 2019 EU guidelines on the ethics of AI raised concerns about the regulation of digital governance among other trepidations [3]. It is widely accepted that digital healthcare and AI will forever change the ways in which healthcare is delivered to patients, as well as the relationship between doctors and patients. There are multiple objectives of digital health services: achieve cost efficiency gains, improve the effectiveness of medical treatments, boost the early diagnosis of illnesses, enhance surgeries with robotic systems, increase positive healthcare outcomes, and help good governance of the public sector by enhancing transparency and accountability [4].

This article aims to take stock of the opportunities offered by digital technologies within the healthcare sector against the backdrop of structural bureaucratic rigidities and organizational resistance to embracing innovation. Some have argued that AI has triggered a process of “innovative disruption”, because it will radically transform the healthcare model from a business-to-business model to a business-to-consumer model [5]. In these new systems, doctors will no longer be the bosses. In a recent article, Richard Saltman discussed the impact of the digital revolution (referring mainly to computer technology) on the tax-funded healthcare systems of Europe. His primary focus was on the future challenges and organizational constraints that will create obstacles to the successful implementation of digital health governance [6]. He particularly emphasized the factors that determine the slow progress of organizational innovation when implementing digital technologies and new integrated delivery arrangements within European tax-funded healthcare systems. He criticized the organizational stasis of public sector organizations, which he labelled as a structural blockage, citing resistance from public hospitals to organizational changes, internal reforms, and innovation. In regards to AI, the fear is that robotic systems will replace doctors when it comes to performing healthcare diagnoses. For instance, retinal scans and targeted radiotherapy use AI systems to carry out diagnoses with increasing confidence. Doctors will need to be trained in data science and upgrade their technology skills to be able to “control” these systems. However, the fear of job losses is exaggerated. In fact, AI is likely to create new jobs, for instance, in the field of machine learning and data science analysis.

The article by Richard Saltman pointed to the need for more dynamic organizational change within public sector hospitals. Saltman also offered an insightful discussion of some of the best practices of service delivery in Europe and how they succeeded in overcoming the tendencies towards inertia and ultimately embraced the digital transformation of service delivery. We need to take a step back and further elaborate on what “bureaucratic inertia” means in the context of European healthcare services. The issue of change in a bureaucratic system is often framed in dysfunctional terms (see, for instance, the term “disruption”), emphasising the traits of public administration that lead inevitably to a pathological state of institutional inefficiency and malaise, a so-called “vicious cycle” in Crozier’s terms [7].

Saltman’s article poses the important question of distinguishing between “dynamic” and “stable” healthcare systems, attributing to the former the capacity to innovate and to the latter a sort of pathological immobilisme. European tax funded systems belong to the latter category. In many European healthcare systems, administrative and managerial change does not happen in big and radical steps, and is only feasible through small, incremental reforms that allow for institutional learning and effective implementation [8]. Incrementalism is not very different from inertial change, which refers to a uniform rate of motion done in a predictable series of increments [9]. This definition of inertia emphasises the cumulative effect of incremental changes. Inertia is conceptualised as a “force in motion”, which Richard Rose describes as a “juggernaut”. Despite the legacy of past decisions, incremental changes made by governments can cumulatively produce big changes in the end [10]. Digital healthcare is likely to be adopted in incremental steps within tax-funded European systems. On one hand, technology keeps an eye on patients and is often in the background of medical data and patient experiences. On the other hand, AI is technically less developed and less autonomous from human intelligence than we assume. Further technological advances are needed to develop “strong” systems of AI [11]. Furthermore, digital healthcare is increasingly subject to regulatory governance by national and international bodies, while ethical concerns are, for good or for worse, significantly slowing down the pace of AI and other technologies.

## The path to digital innovation: from information technology (IT) to patients’ empowerment through artificial intelligence (AI)

Technology, particularly digital technology, is increasingly playing a key role in leveraging future health policies. In the last decade, national governments in Europe have designed and implemented policies aimed at restructuring the delivery of healthcare services by using new digital health infrastructures and services [12]. Electronic health records and patient data have been at the core of this digitization process. Artificial Intelligence (AI) requires medical health data, which is the most valuable asset for AI producers and companies. Healthcare data is not merely a preference for marketing purposes, but refers to symptoms, treatments, and patient experiences. In fact, this term refers to highly sensitive patient data protected by national legal systems and regulations.

In the earlier stages on the path towards improved digital healthcare, governments used ICT (Information and Communication Technologies) to manage internal administrative processes. The main purpose of digitization was cost efficiency and improved effectiveness of procedures. The administration of health units has been using ICT for reimbursement procedures and e-procurement, for instance. However, digitization in the early days did not entail the participation of users and patients in the process of service delivery.

More recently, e-health and e-government policies have substantially redesigned the role of communication with patients and their relatives [13]. These policies have allowed for the development of more user-friendly, personalized services that rely on digital technologies, such as the use of smartphone applications to monitor patients’ health conditions. On the path towards digital governance, the use of digital technologies is now moving beyond the back office of administrative processes and health data collection, and being consistently applied to direct service delivery and direct citizen engagement. For instance, NHS Highland recently launched a new programme that uses virtual reality to help patients prepare for upcoming MRI scans in Scotland. This virtual device simulates the MRI experience for patients, thus helping them be more comfortable during the procedure and making the MRI more bearable. This is just one example of how AI improves the experience of patients.

Our future challenge is not merely to introduce ICT to public organizations, but also to reframe the traditional relationship between professionals and patients through the use of data sharing and digital technologies. Thus, the changes affecting healthcare systems will be transformative and not just marginal. The doctor-patient relationship will change, which has given rise to legitimate concerns. The empowerment of patients through digital platforms has been adopted in order to promote self-management of diseases and improve patients’ engagement in the decision making process [14]. Digital platforms allow patients and their families to have access to a wealth of information about their conditions and to connect more easily with professionals. The use of digital platforms provides the tools for data-based personalization of medicine and significantly increases the focus on the customer and their specific needs [15].

However, as AI takes over low-level work, such as monitoring heart rate or sleeping patterns, there are concerns about the loss of human touch, which is so integral to the patient-doctor relationship. It is possible that AI will serve as a gatekeeper and patients will interact with a machine for low-level and routine practices. Despite the possible costs of losing some human touch for these services, AI will free up more time for doctors to treat patients who need specialized care. For instance, Ichilov Hospital in Tel Aviv, one of the largest hospitals in Israel, launched a medical trial in September 2019 to remotely monitor patients’ conditions using sensors and cameras. The trial used an early detection instrument for patients’ deteriorating conditions, which reduced the nurses’ workload significantly. Ichilov Hospital collaborated with two Israeli AI companies, which specialize in monitoring systems (Innovision and BioBeat). In 2019, the Israeli government launched a massive reform plan to unleash AI technologies in healthcare organizations, making use of the country’s advances in cyber security technology.

In the field of oncology, digital healthcare has increased the confidence of early stage diagnosis. For instance, it provides quicker, better, and more cost-efficient image analysis of prostate biopsies. Precision medicine and new technologies promise to support doctors in matching the immunoprofiles of patients using targeted drugs for individual patients [16]. Robotic systems are used effectively and widely for complex surgeries. Another example of a widely used digital technology is IBM’s Watson, an expert system developed by the technological giant IBM. Watson is used as an instrument for doctors to identify individualized, evidence-based treatments. It collects a huge amount of medical data and presents doctors with treatment options, then recommends personalized drugs. The fees range from $200 to $1000 USD per patient.

## Hybrids and accountability

Saltman’s article does not discuss the benefits of digital healthcare in terms of precision and personalized medicine. Instead, the piece is more concerned with the organizational impact of information technologies on public sector providers and public hospitals. In his article, Saltman advocates greater structural diversity within European NHS systems and better links with intermediate non-public providers and non-governmental actors. This is a highly contested proposal for tax-funded Beveridge healthcare systems, for which public hospitals are central elements of accountability for public health and wellbeing [17]. Their institutional objectives depend on equity and universal access-oriented efforts. The majority of hospitals are still publicly owned and managed, despite a wide range of managerial reforms and leadership changes over the last few decades [18].

Major organizational changes began in the late 1980s in Sweden and England, due to growing political pressure on public authorities to restructure the hierarchal governance models of healthcare providers [19]. According to Richards and Smith, new forms of hospital governance pushed the state to focus on directing and regulating rather than managing hospitals [20]. This meant, for instance, the establishment of new public-private partnerships between different stakeholders. In these arrangements, the private sector is involved in delivering public goods and services and serves as a vehicle for coordinating with non-governmental actors in order to undertake integrated efforts to meet healthcare needs [21]. The post-2008 economic crisis in Europe reinforced the shifting role of the state from rowing to steering [22]. As a result, we now have a diverse provider environment within the European NHS systems, as well as hybrid forms of accountability [23] and performance measurement systems [24]. AI producers and developers are for-profit businesses in many countries, which provide products and services to public sector organizations. Traditional mechanisms of accountability need to adjust to the new AI environment, which is characterized by multiple stakeholders sharing medical data, research, and systems.

The new digital environment for healthcare services requires collaborative infrastructures and partnerships across different stakeholders in the public, private, and not-for-profit sectors. Hybrid forms of management have emerged and will only continue to grow [25]. For instance, data sharing agreements between different providers are central to AI systems. Hybrid accountability describes the integration of accountability arrangements between and across public, market, and social accountability regimes. Hybrid forms of accountability layer and mix actors, logics, norms, and mechanisms from public, market, and social accountability regimes by concurrently applying two or more of these differing regimes to the same situation [26].

## Ethical concerns and regulations

We need clearer guidance about the accountability of AI, in order to foster an effective regulatory environment that does not stifle innovation and govern hybrid forms of arrangements without eschewing human agency and oversight. This is probably the most critical concern associated with digital healthcare in the future. Regulation is indeed necessary to protect patients’ safety, privacy, and fundament rights and freedoms. As with the pharmaceutical industry, AI developers should be regulated by licensing and critical appraisal to remove unsafe systems and procedures. A delicate balance needs to be found between fostering innovation and regulating the environment to implement AI in healthcare [27].

On the one hand, digital healthcare will disrupt the existing delivery of healthcare services, enhancing a redesign of internal organizational systems and procedures for public accountability and transparency. For instance, medical data storage associated with machine learning is subject to new legal frameworks and risk assessment obligations, such as the data protection impact assessment [28]. Hospitals and health organizations must revise internal procedures and design new employee training programs to comply with European data privacy regulations. Machine learning is particularly opaque, thus it is highly difficult to ensure transparency about its use and deployment. The EU ethical guidelines recommend that AI systems should be identifiable and humans need to be aware of the content of their interactions with machines. Furthermore, patients need to be able to understand how AI systems work and how these technologies can be applied to their conditions. Transparency and accountability requirements are changing the governance systems of health organizations.

NHS healthcare systems in Europe have adopted and deployed artificial intelligence (AI), machine learning, big data analytics, wearables, and other new technologies to improve clinical accuracy, patient safety, precision medicine, and personalized medicine. Yet, government regulations may risk slowing down rapid developments over concerns for accountability and the protection of fundamental human rights. The EU is a frontrunner in this policy area, as well as the ethical guidelines regarding AI. The European Parliament has expressed many concerns about the use of patients’ data and the security of data generated through AI. In 2017, they initiated actions to legislate and regulate the new AI environment. Digital healthcare certainly poses new challenges to accountability arrangements that had previously been based on a (human) principal and (human) agents. The old arrangements of accountability will certainly be challenged by AI, given the ethical and philosophical implications associated with clinical choices and the ranking of options, not made by humans. The EU guidelines recommend that clinical decisions rest in the hands of doctors and that machines should be subject to human oversight. Some scholars argue that a new concept of information accountability is necessary to manage these processes [29].

For instance, what happens when IBM’s Watson for Oncology (or other expert systems described earlier in this article) is simply wrong? Legal frameworks associated with medical malpractice apply to people (the medical professional or organization), but not machines. Existing legal frameworks in many countries are not prepared for sanctioning errors by machines that make mistakes whilst establishing a treatment ranking or identifying drug prescriptions for individual patients. Personhood is the legal basis for liability concerning malpractice in both civil and tort law. What happens when a machine makes an error that has detrimental consequences for individual patients? Clearly a machine cannot be in full control of itself. Thus, it is essential that regulation provides for human oversight of these technologies and that doctors remain in charge of clinical decision-making.

The EU has promoted a “human centric-approach” to AI that centrally focuses on the respect of fundamental rights, including those set out in the Fundamental Rights of the European Union. In April of 2019, the EU Commission drew up non-binding guidelines for AI design, development, deployment, and implementation. The thrust of this ethical approach is to protect human dignity and agency throughout the use of these new technological systems by ensuring human control when interacting with AI. Other ethical principles set out in the EU Commissions’ guidelines are privacy and data protection to ensure that citizens are always in full control of their own data. Thus, AI systems in medical care should be designed with techniques, such as data encryption and anonymisation to protect patient data. Although the EU is a global frontrunner in setting principles for the AI community (including for giants such a Google, IBM, and Microsoft), the EU ethics guidelines thus far are not legally binding and the regulatory oversights that support their implementation are weak. A 2019 study by the European Parliament recommends the creation of a regulatory body for algorithmic decision-making, which will handle specific liability and certification regimes [30].

To conclude, tax-funded European healthcare systems operate in a provider environment that is much more organizationally diverse than it was 20 years ago. Partnership arrangements with non-governmental actors and civil society are today an established governance model in many countries. Hybrid organizational forms that mix private and public delivery systems are the result of waves of internal organizational restructuring [31]. AI will consolidate the process of hybridization in healthcare governance. National governments in Europe and the European Commission have embraced digital healthcare with enthusiasm, followed by legislative impetus and regulatory frameworks that improve patients’ dignity, transparency of governance processes, and enhance patient engagement. The pace of change may still appear slow to observers in other parts of the world, but one should not be misguided by this slow rate of progress. Major change is in the works, even though it is advancing slowly. The pace of change is a result of the orientation of NHS healthcare systems towards values of equity, universal access, and organizational values that are fundamentally different from those of privatized medicine. Changes are incremental because of the desire to align the population’s health needs with equity, efficiency, and safety. Digital governance is widely accepted as the way to achieve these objectives. The pace of change will remain uncertain because new hybrid accountability regimes will need to be designed and AI will need to be regulated at the national and European level. The outcome will be a transformative change that will integrate digital technologies into public sector modernization. AI has great potential to empower patients, while improving their health, personalized experiences, and individual wellbeing.
